# Iron-based metal-organic framework co-loaded with buthionine sulfoximine and oxaliplatin for enhanced cancer chemo-ferrotherapy via sustainable glutathione elimination

**DOI:** 10.1186/s12951-023-01998-w

**Published:** 2023-08-10

**Authors:** Zhiping Rao, Yutian Xia, Qian Jia, Yutong Zhu, Lexuan Wang, Guohuan Liu, Xuelan Liu, Peng Yang, Pengbo Ning, Ruili Zhang, Xianghan Zhang, Chaoqiang Qiao, Zhongliang Wang

**Affiliations:** 1 Lab of Molecular Imaging and Translational Medicine (MITM), Engineering Research Center of Molecular & Neuroimaging, Ministry of Education, School of Life Science and Technology, Xidian University & International Joint Research Center for Advanced Medical Imaging and Intelligent Diagnosis and Treatment, Xi’an, 710126 Shaanxi China; 2 Academy of Advanced Interdisciplinary Research, Xidian University, Xi’an, 710071 Shaanxi China; 3 State Key Laboratory of Molecular Vaccinology and Molecular, Diagnostics & Center for Molecular Imaging and Translational Medicine, School of Public Health, Xiamen University, Xiamen, 361102 China

**Keywords:** Metal-organic frameworks, Ferroptosis, GSH elimination, Cancer therapy

## Abstract

**Background:**

Emerging ferroptosis-driven therapies based on nanotechnology function either by increasing intracellular iron level or suppressing glutathione peroxidase 4 (GPX4) activity. Nevertheless, the therapeutic strategy of simultaneous iron delivery and GPX4 inhibition remains challenging and has significant scope for improvement. Moreover, current nanomedicine studies mainly use disulfide-thiol exchange to deplete glutathione (GSH) for GPX4 inactivation, which is unsatisfactory because of the compensatory effect of continuous GSH synthesis.

**Methods:**

In this study, we design a two-in-one ferroptosis-inducing nanoplatform using iron-based metal-organic framework (MOF) that combines iron supply and GPX4 deactivation by loading the small molecule buthionine sulfoxide amine (BSO) to block de novo GSH biosynthesis, which can achieve sustainable GSH elimination and dual ferroptosis amplification. A coated lipid bilayer (L) can increase the stability of the nanoparticles and a modified tumor-homing peptide comprising arginine-glycine-aspartic acid (RGD/R) can achieve tumor-specific therapies. Moreover, as a decrease in GSH can alleviate resistance of cancer cells to chemotherapy drugs, oxaliplatin (OXA) was also loaded to obtain BSO&OXA@MOF-LR for enhanced cancer chemo-ferrotherapy in vivo.

**Results:**

BSO&OXA@MOF-LR shows a robust tumor suppression effect and significantly improved the survival rate in 4T1 tumor xenograft mice, indicating a combined effect of dual amplified ferroptosis and GSH elimination sensitized apoptosis.

**Conclusion:**

BSO&OXA@MOF-LR is proven to be an efficient ferroptosis/apoptosis hybrid anti-cancer agent. This study is of great significance for the clinical development of novel drugs based on ferroptosis and apoptosis for enhanced cancer chemo-ferrotherapy.

**Graphical Abstract:**

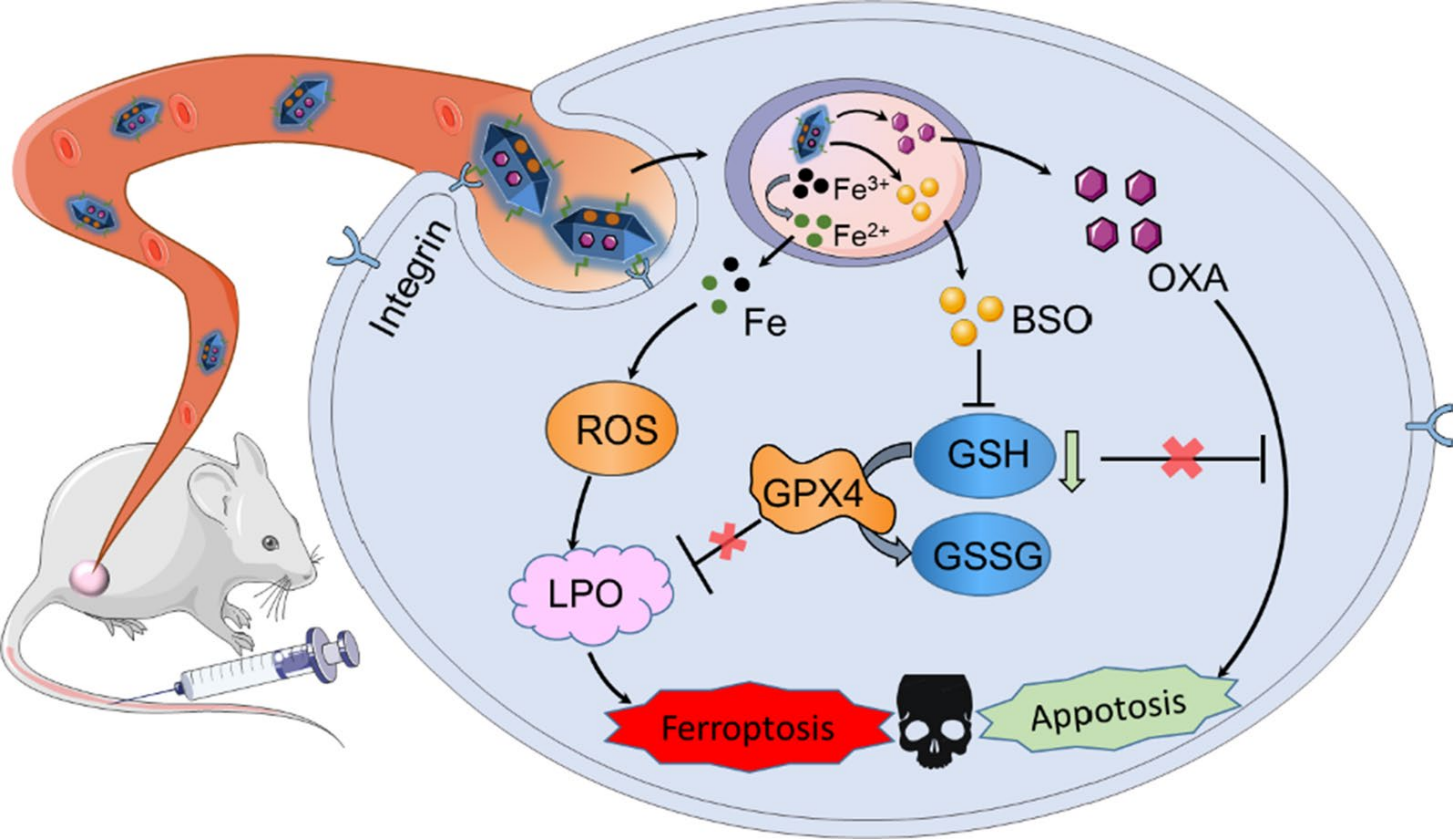

**Supplementary Information:**

The online version contains supplementary material available at 10.1186/s12951-023-01998-w.

## Introduction

Cancer is one of the leading causes of mortality worldwide, and the existing therapeutic approaches for cancer are still far from satisfactory. Therefore, there is an imperative need to integrate emerging biomedical discoveries and technological innovations with conventional therapies. Ferroptosis, a newly discovered non-apoptotic form of cell death [1–3], is characterized by the iron-dependent generation of reactive oxygen species (ROS) and the accumulation of lipid peroxides (LPO) [4, 5]. The distinctive metabolism of cancer cells, their specific mutations with regard to certain tumor suppressors or oncogenes, the imbalances in terms of ferroptosis defenses, and high ROS loads render some tumor cells vulnerable to ferroptosis [6–8], which has been proven to be an effective method for cancer treatment.

Many strategies have been established to induce ferroptosis in cancer cells [9–11]. In particular, iron-based nanomaterials, such as iron oxide nanoparticles [12–14], amorphous iron nanoparticles [15], and FePt nanoparticles [16, 17] have been widely used to increase cellular iron level and trigger the Fenton reaction for generating ROS and LPO. However, some tumor cells can use glutathione peroxidase 4 (GPX4), a lipid repair enzyme, to decrease LPO accumulation by converting toxic LPO to non-toxic lipid alcohols in the presence of glutathione (GSH), thereby protecting themselves from ferroptosis [18, 19]. Therefore, to achieve effective ferroptosis in cancer cells, it is important to inactivate GPX4 or deplete GSH while delivering iron. Nevertheless, achieving this goal efficiently with iron oxide or amorphous iron nanoparticles remains challenging because of their limited drug-loading capabilities. Hence, there is an urgent need to construct a multifunctional nanoplatform that can not only deliver a high dose of iron but also inactivate GPX4 or consume GSH to achieve efficient ferroptosis induction.

Iron-based metal-organic frameworks (MOFs) have garnered considerable attention owing to their high biocompatibility, tunable surface modification, and high drug loading capacity [20, 21]. In a previous study of ours, we successfully developed a rabies virus-inspired iron-based MOFs for targeted imaging and treatment of glioma [22], taking advantage of the simple and economical synthesis method for these substances can achieve satisfactory control of the size and high drug loading for ferroptosis induction. The iron released from the MOFs bone structure can trigger the Fenton reaction and induce ferroptosis, and the ultra-high drug-loading capacity of MOFs permits multiple drug encapsulation to further enhance ferroptosis. Studies have demonstrated the superiority of iron-based MOFs for ferroptosis-mediated tumor therapy [23–26]. Nevertheless, these studies mainly used disulfide-thiol exchange to deplete GSH for GPX4 inactivation, which is difficult to inactivate GPX4 completely because of the compensatory effect of the continuous synthesis of GSH. In this study, we used a small molecule, buthionine sulfoxide amine (BSO), to block de novo GSH biosynthesis and eliminate it completely. BSO is less cytotoxic than other agents, because it cannot trigger ferroptosis in breast cancer cells on its own [27, 28]. Therefore, we designed a dual ferroptosis amplification strategy using an iron-based MOFs that combined iron supply and de novo GSH elimination for effective ferroptosis management.

**Scheme 1.**
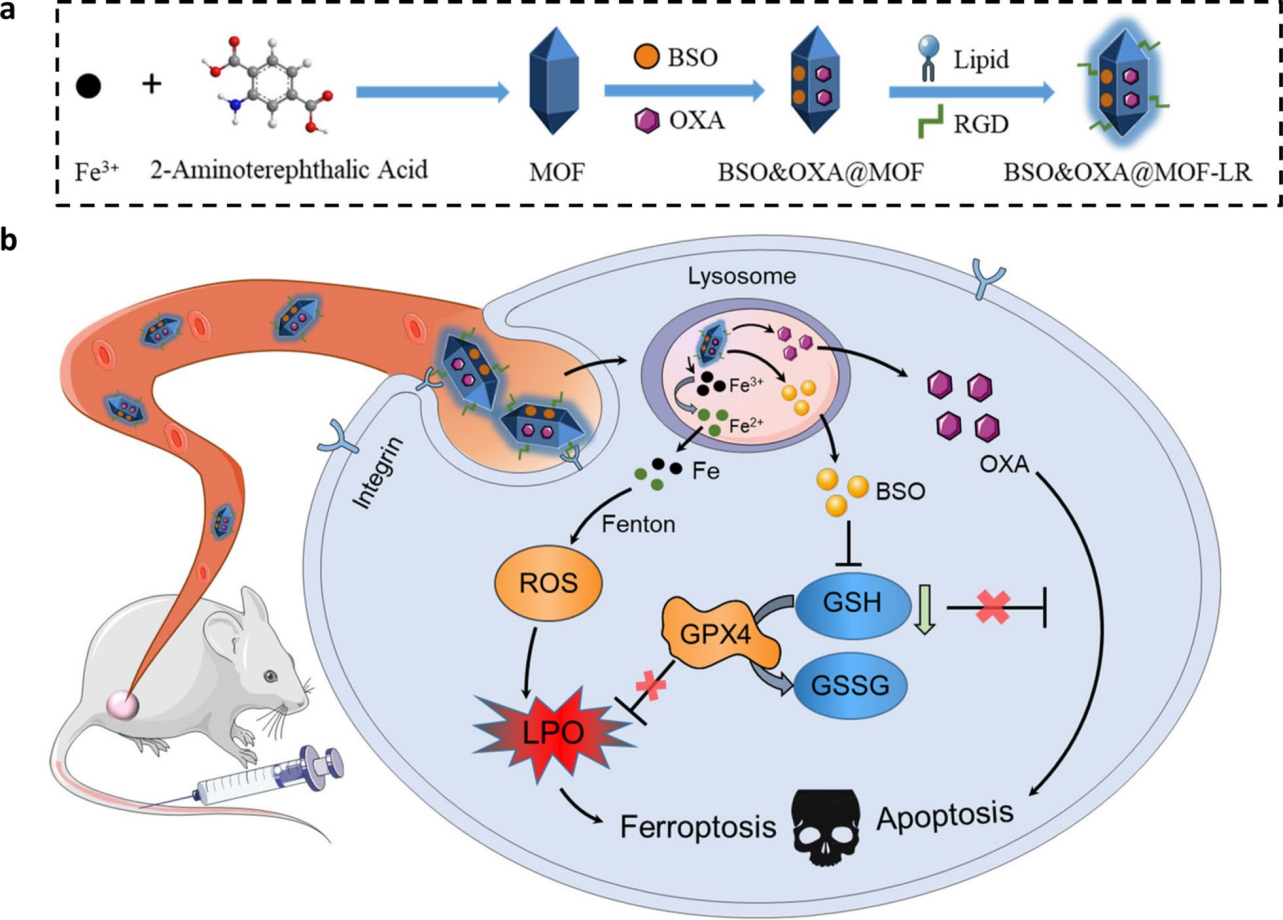
Schematic illustration of the preparation of BSO&OXA@MOF-LR for ferroptosis/apoptosis-based cancer therapy. **a** Synthesis of BSO&OXA@MOF-LR. **b** The mechanism of BSO&OXA@MOF-LR action for tumor therapy, which is achieved by inducing both ferroptosis and apoptosis. *MOF* metal-organic framework; *BSO* buthionine sulfoxide amine; *OXA* oxaliplatin; *RGD* arginine-glycine-aspartic acid; *ROS* reactive oxygen species; *GPX4* glutathione peroxidase 4; *GSH* glutathione; *GSSG* oxidized glutathione

We first synthesized an iron-based MOFs loaded with BSO and then modified it with a lipid bilayer (BSO@MOF-L) using a simple and economical synthesis method. Once the nanoparticles were internalized by cancer cells, the ferric ions released from the bone structure of the MOFs could be reduced by metal reductase or GSH and then triggered the Fenton reaction to produce ROS, and high ROS concentrations caused severe lipid peroxidation. The released BSO could inhibit the synthesis of GSH, leading to the inactivation of GPX4 and causing further accumulation of LPO. Moreover, the coated lipid bilayer was able to increase the stability of the nanoparticles in the bloodstream, which was crucial for maintaining their structural integrity such that they could execute their function in vivo. To enhance tumor targeting of nanoparticles, a tumor-homing peptide comprising arginine-glycine-aspartic acid (RGD/R) was also introduced (BSO@MOF-LR) for tumor-specific therapy. In summary, this study used a simple and easily synthesizable multifunctional nanoplatform to effectively target breast cancer cells via a dual ferroptosis amplification strategy including sufficient iron supply and de novo GSH inhibition (Scheme 1).

In addition, some studies have found that the resistance to oxaliplatin (OXA) in cancer cells is partially due to increased GSH content [29, 30]. Therefore, we speculated that GSH depletion might be capable of alleviating OXA resistance and enhancing its chemotherapeutic efficacy in cancer cells. Accordingly, we also loaded OXA onto our newly-designed nanodrug, to obtain BSO&OXA@MOF-LR, for combined cancer therapy. In addition to ferroptosis, decreased GSH level promote the induction of apoptosis by OXA. Therefore, the BSO- and OXA-based nanodrug BSO&OXA@MOF-LR was designed to conduct a hybrid ferroptosis/apoptosis anticancer therapy (Scheme 1). This strategy may provide a new perspective for improving the outcomes of traditional cancer therapies.

## Results and discussion

### Preparation and characterization of BSO@MOF-L

**Fig. 1:**
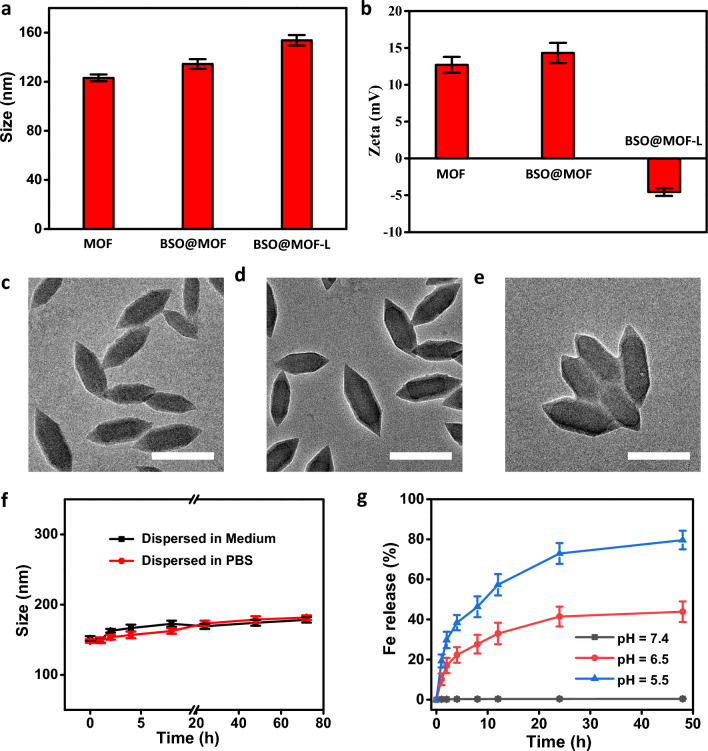
Characterization of the MOF nanoparticles. **a** Hydrodynamic size of MOF, BSO@MOF, and BSO@MOF-L. **b** Zeta potential of MOF, BSO@MOF, and BSO@MOF-L (n = 3). **c-e** Transmission electron microscopy images of MOF, BSO@MOF, and BSO@MOF-L (scale bar: 150 nm). **f** Changes in size of BSO@MOF-L in PBS or culture medium, indicating good stability. **g** The release of iron from BSO@MOF-L at different pH values. *MOF* metal-organic framework; *BSO* buthionine sulfoxide amine; *PBS* phosphate-buffered saline

To verify the successful induction of ferroptosis, we first synthesized BSO@MOF-L. The MOF skeleton was prepared using a simple method, according to a previously reported protocol [22]. BSO was then loaded into the MOF pores through adsorption, followed by modification of the lipid bilayer using a two-step approach [22]. The synthesized MOF nanoparticles exhibited good dispersibility with average hydrodynamic size of approximately 120 nm, which was similar to previous reports by the Zhao and Meng research groups [31, 32]. The hydrodynamic size did not change significantly after drug loading (BSO@MOF) and had a slight increase after lipid modification (BSO@MOF-L, approximately 150 nm) (Fig. 1a). Zeta potential measurement indicated a positive zeta potential of + 14.67 mV for BSO@MOF, which was consistent with previous work [33]. The zeta potential changed from positive to negative after lipid modification, indicating that the 1,2-dioleoyl-sn-glycero-3-phosphocholine was successfully modified on the surface of the BSO@MOF (Fig. 1b). Transmission electron microscopy images of the MOF, BSO@MOF, and BSO@MOF-L nanoparticles displayed a regular bipyramidal rod morphology with a length of approximately 170 nm and a diameter of 70 nm. There were no obvious changes in morphology during drug loading and lipid modification (Fig. 1c–e). Additionally, the final BSO@MOF-L showed good stability in both phosphate-buffered saline and cell culture medium within 72 h (Fig. 1f). For efficient ferroptosis induction, the release of iron at the tumor site is critical. Therefore, we also estimated the iron release from BSO@MOF-L at different pH values. There was no iron release under neutral conditions (pH 7.4), whereas the release was up to 70% at 24 h at a pH 5.5, which was much higher than the release at pH 6.5 (40%), implying a tumor microenvironment response and sufficient release of iron (Fig. 1g). All these results suggested successful synthesis of the BSO@MOF-L nanoparticles.

### BSO@MOF-L induces tumor cell death via ferroptosis

**Fig. 2:**
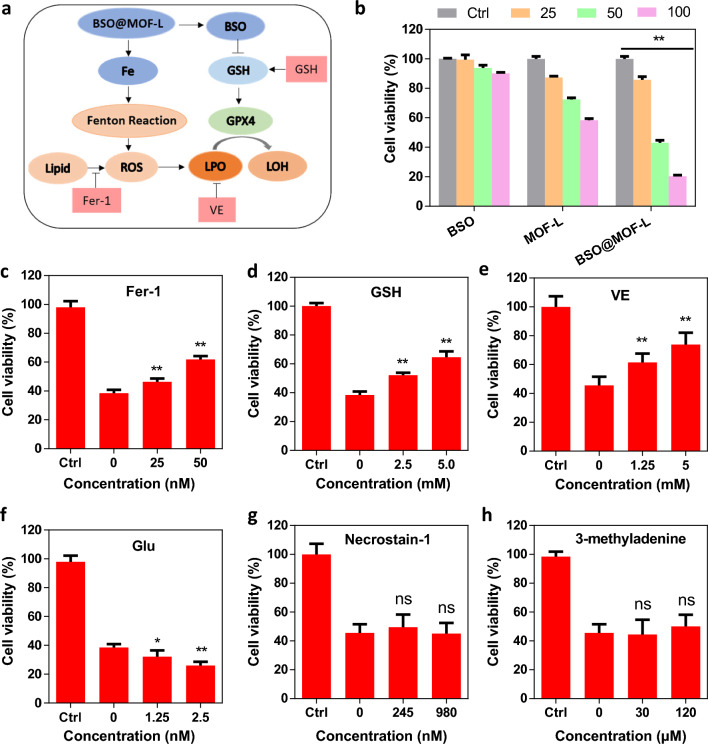
Evaluation of the mechanism of BSO@MOF-L-induced cell death. **a** Schematic illustration of ferroptosis and related ferroptotic regulators. **b** Cell viability of 4T1 cells after treatment with different concentrations of the nanoparticles (n = 4). **c–h** Cell viability of 4T1 cells after treatment with BSO@MOF-L and **c** Fer-1, **d** GSH, **e** VE, **f** Glu, **g** Necrostain-1, or **h** 3-methyladenine (**c**–**f**, n = 5; g-h, n = 4). * *p* < 0.05, ** *p* < 0.01. Fer-1, ferrostatin-1; *GSH* glutathione; *VE* vitamin E; *Glu* glutamate; *ROS* reactive oxygen species; *LPO* lipid peroxides; *GPX4* glutathione peroxidase 4; *LOH* lipid alcohols

After the successful preparation of BSO@MOF-L, the cell-killing effect of the nanoparticles and the underlying mechanisms were explored (Fig. 2a). First, we conducted cytotoxicity experiments using the mouse breast cancer cell line 4T1. The results showed that free BSO was essentially non-toxic to 4T1 cells. The cell viability was over 90% even at a high BSO concentration of 100 μg mL^−1^, indicating that although BSO acted as a ferroptosis inducer, it could not induce cell death in 4T1 cells on its own (Fig. 2b), which was consistent with previous work [27, 28]. This result maybe due to BSO cannot induce the accumulation of LPO, so it cannot cause LPO-mediated cell death. Moreover, simply release of iron from MOF-L was insufficient to induce cell death. Interestingly, when BSO and MOF-L were combined to obtain BSO@MOF-L, we observed a significant cell-killing effect, with the cell viability decreasing to 25% at 100 μg mL^−1^ BSO@MOF-L (Fig. 2b). We further tested the viability of 4T1 cells after treatment with different concentrations of BSO@MOF-L at various time points. The results showed that cell viability significantly decreased with increasing concentrations of BSO@MOF-L, but not with time lapse (Additional file 1: Fig. S1). Therefore, we treated 4T1 cells with 100 μg mL^−1^ of BSO@MOF-L for 24 h in our subsequent experiments.

To verify the occurrence of ferroptosis, different regulators of ferroptosis, necrosis, autophagy, and apoptosis were used to regulate the viability of the BSO@MOF-L-treated cells. The cell viability was significantly increased upon co-delivery of BSO@MOF-L with ferroptosis inhibitors, such as ferrostatin-1, GSH, and vitamin E, which acted by radical-trapping or antioxidation (Fig. 2c–e). Glutamate promoted cell death because cellular intake of cysteine relies on the exchange involving an intracellular glutamate efflux (Fig. 2f). However, the necrosis inhibitor necrostain-1, the autophagy inhibitor 3-methyladenine, and the apoptosis inhibitor Z-VAD-FMK could hardly rescue 4T1 cells from death (Fig. 2g, h, Additional file 1: Fig. S2). All these results indicated that there was few necrosis, autophagy or apoptosis, and the main cell death mode was ferroptosis.

**Fig. 3:**
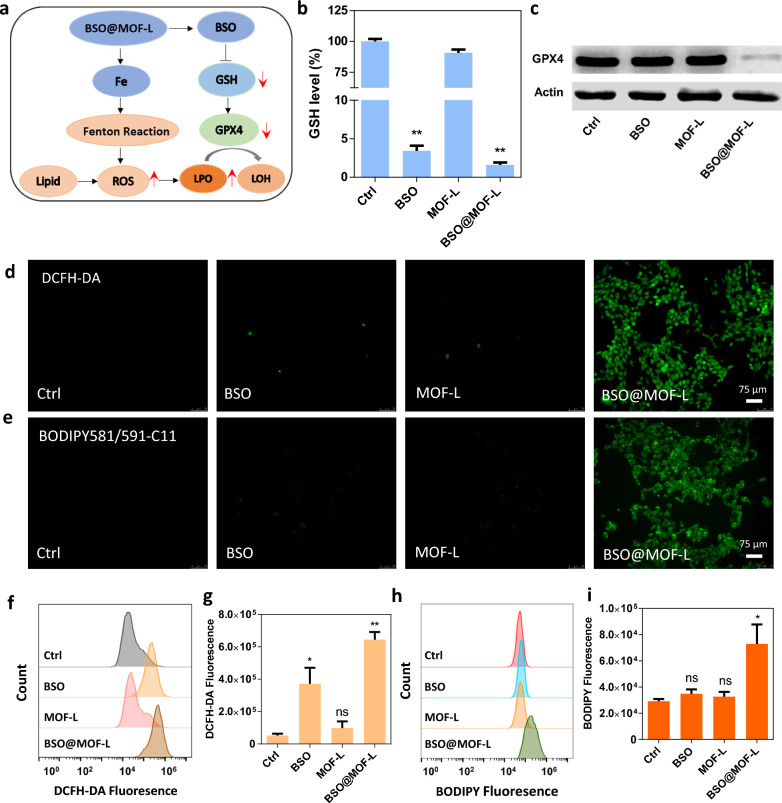
Analyses of the cellular factors involved in BSO@MOF-L-mediated ferroptosis. **a** Schematic illustration of the ferroptosis mechanism. **b** Intracellular GSH level and **c** GPX4 expression of 4T1 cells after treated with BSO, MOF-L, or BSO@MOF-L. Microscopic images of cells after DCFH-DA (**d**) and BODIPY581/591-C11 (**e**) staining after treated with BSO, MOF-L, or BSO@MOF-L. Flow cytometry analysis of the 4T1 cells after DCFH-DA **f**–**g** and BODIPY581/591-C11 **h**–**i** staining with different treatments (n = 3). DCFH-DA, dichlorofluorescein diacetate. * *p* < 0.05

We hypothesized that BSO@MOF-L could efficiently inhibit the synthesis of GSH, a substrate of GPX4 for antioxidants, to inhibit LPO accumulation (Fig. 3a). Accordingly, we analyzed intracellular GSH inhibition. The GSH level in 4T1 cells treated with free BSO or BSO@MOF-L were significantly lower than that of control and MOF-L groups, demonstrating the successful inhibition of GSH synthesis by BSO (Fig. 3b). The GPX4 activity decreased in the BSO@MOF-L group, as indicated by the changing amount of nicotinamide adenine dinucleotide phosphate over time (Additional file 1: Fig. S3). Western blot analysis was used to further test the expression of GPX4 in 4T1 cells. Compared with the control and the treatment with BSO or MOF-L, BSO@MOF-L effectively down-regulated the GPX4 level inside cells (Fig. 3c). Next, the intracellular levels of ROS and LPO, which are important hallmarks of ferroptosis, were measured following the different treatments. ROS levels were significantly elevated after treated with BSO@MOF-L, as determined by dichlorofluorescein diacetate staining followed by microphotography (Fig. 3d) and flow cytometry analysis (Fig. 3f, g), both of which indicated that the highest generation of ROS occurred in BSO@MOF-L-treated cells. The lipid peroxidation sensor BODIPY-C11 was used to evaluate LPO generation by light microscopy (Fig. 3e) and flow cytometry analysis (Fig. 3h, i). As shown in Fig. 3e, cells in the BSO@MOF-L group exhibited a stronger fluorescence intensity than those in the other groups. These results demonstrated that our nanoparticles could cause ROS and LPO generation, ultimately triggering ferroptosis.

### Tumor targeting of the RGD-modified nanoparticles

**Fig. 4:**
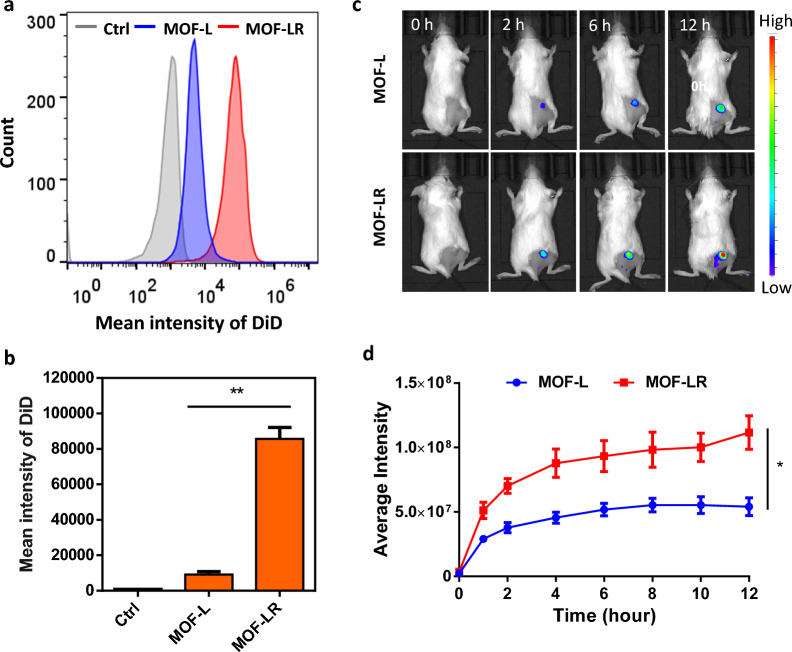
Tumor targeting of RGD-modified nanoparticles. **a-b** Analysis of the cellular uptake of MOF-L and MOF-LR by flow cytometry (n = 3). **c** The biodistribution of MOF-L and MOF-LR in vivo. **d** Average fluorescence intensity of DiR in the tumor tissue is higher over time in the MOF-LR group than that in the MOF-L group. * *p* < 0.05, ** *p* < 0.01

To further investigate in vivo targeted tumor therapy, we modified MOF-L with RGD (MOF-LR) and tested whether the modification could enhance the cellular uptake of the nanoparticles. MOF-L and MOF-LR were labeled with the lipophilic dye 1,1-dioctadecyl-3,3,3,3-tetramethylindodicarbocyanine (DiD) and incubated with 4T1 cells for 2 h. Cellular uptake was then analyzed by flow cytometry. The results showed an enhanced fluorescence intensity of DiD-labeled MOF-LR compared to that of MOF-L (Fig. 4a, b). Using an in vivo fluorescence imaging system, rapid accumulation of red fluorescent signal was detected in the tumor site 2 h post-injection of 1,1-dioctadecyl-3,3,3,3-tetramethylindotricarbocyanine iodide (DiR)-labeled MOF-LR (Fig. 4c). The DiR fluorescence intensity in the tumor site of the MOF-LR group was stronger than that of the MOF-L group (Fig. 4d), indicating a successful tumor-targeting modification that was suitable for in vivo experiments.

### In vivo anti-tumor efficacy

**Fig. 5:**
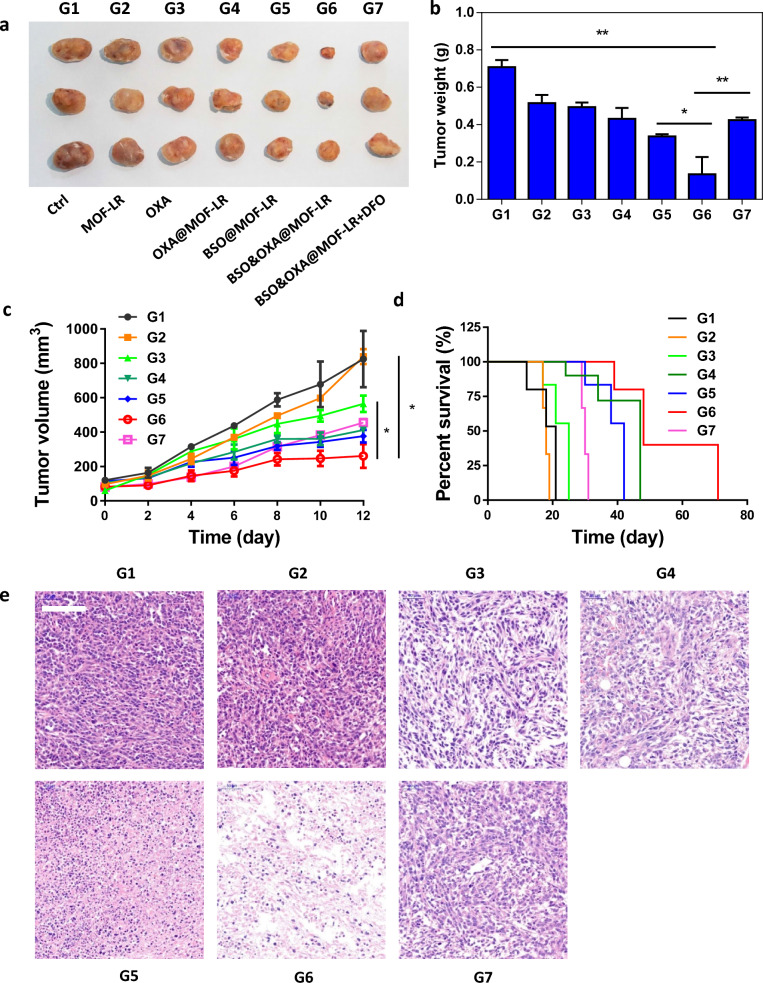
In vivo anti-tumor efficacy of BSO&OXA@MOF-LR. **a** Photographs of tumors obtained on day 12 after various treatments.** b** Quantitative analysis of tumor mass 12 days after different treatments (n = 3).** c** Tumor growth curve of different groups (n = 3). **d** Survival curve of mice after they received different treatments (n = 3). **e** Representative hematoxylin and eosin staining images of tumor slices at the end of the efficacy experiment (scale bar: 100 μm). * *p* < 0.05, ** *p* < 0.01. *OXA* oxaliplatin; *DFO* deferoxamine

Studies have demonstrated that decreased GSH levels can alleviate resistance to chemotherapy drugs and enhance chemotherapeutic efficacy. For this reason, we also loaded OXA onto our nanodrug, to obtain BSO&OXA@MOF-LR for combined tumor therapy. The hydrodynamic size and zeta potential of BSO&OXA@MOF-LR was very similar to that of BSO@MOF-L (Fig. 1a, b, Additional file 1: Fig. S4). The cell-killing effect of BSO&OXA@MOF-LR was markedly increased compared to that of BSO@MOF-LR or OXA@MOF-LR, indicating the synergistic effect of ferroptosis inducer and chemotherapeutic drug (Additional file 1: Fig. S5). The in vivo therapeutic efficacy of both BSO@MOF-LR and BSO&OXA@MOF-LR was evaluated in 4T1 tumor xenograft mice. The nanodrugs were administered intravenously when the tumor volume reached approximately 50 mm^3^. After 12 days, tumor in the BSO@MOF-LR group showed suppressed tumor growth compared with that in the free OXA and OXA@MOF-LR groups, owing to iron-based ROS production and BSO-mediated GSH inhibition in the tumor tissue, eventually resulting in BSO@MOF-LR-induced ferroptosis. Meanwhile, the BSO&OXA@MOF-LR group showed the highest tumor suppression rate among all the groups due to ferroptosis and GSH elimination sensitized apoptosis (Fig. 5a–c, Additional file 1: Fig. S6). Moreover, treatment with BSO&OXA@MOF-LR not only inhibited tumor growth in vivo but also significantly improved the survival rate of mice (Fig. 5d), indicating a combined effect of ferroptosis and apoptosis.

The induction of ferroptosis in vivo by BSO&OXA@MOF-LR was further verified by adding a combination of BSO&OXA@MOF-LR and the ferroptosis inhibitor deferoxamine, which resulted in significantly decreased tumor suppression when compared with that of BSO&OXA@MOF-LR (Fig. 5a–d). This implied that ferroptosis played a key role in the mechanism of BSO&OXA@MOF-LR-induced tumor inhibition. Hematoxylin and eosin (H&E) staining of the tumor tissue confirmed more tumor destruction in the BSO&OXA@MOF-LR group (Fig. 5e). We also evaluated in vivo biosafety and found no significant weight changes in any of the treatment groups during the experiment (Additional file 1: Fig. S7). H&E staining also showed minimal lesions in the hearts, livers, spleens, lungs, and kidneys of the mice after the different treatments (Additional file 1: Fig. S8). These findings consistently suggested that BSO&OXA@MOF-LR has a significant therapeutic effect on breast cancer via ferroptosis and apoptosis without overt toxicity, which holds great promise for future clinical research and treatment for breast cancer.

## Conclusion

We designed and synthesised an iron-based MOF nanoplatform BSO@MOF-L, which not only contained enough iron but also loaded a high dose of BSO. The iron could trigger Fenton reaction to generate LPO, while BSO could inactivate GPX4 to lower the rate of LPO reduction, which finally achieved collaborative inhibition of cancer by inducing efficient ferroptosis. By coating the lipid bilayer to obtain stealth properties for escaping endolysosomal uptake, the stability of the BSO@MOF-L nanoparticles was increased, allowing them to efficiently release their payloads into the tumor cells. Collectively, they overcame cellular regulation and delivered high doses of iron and BSO into cancer cells. To enhance the tumor-targeting properties of nanoparticles, a tumor-homing peptide RGD was modified (BSO@MOF-LR) for tumor-specific therapies. Once the nanoparticles were internalized, ferric ions released from the bone structure were able to trigger the Fenton reaction to produce ROS, which resulted in a significant accumulation of LPO. Meanwhile, the release of BSO was able to effectively inhibit the de novo synthesis of GSH, which inactivated GPX4 and further promoted LPO accumulation by blocking the conversion of LPO to lipid alcohols. This two-pronged approach was therefore able to achieve a high rate of ferroptosis. When chemotherapy drug OXA was also loaded into the nanoparticles to obtain BSO&OXA@MOF-LR, the resultant BSO-mediated decrease in GSH allowed OXA to induce apoptosis more efficiently. BSO&OXA@MOF-L was therefore proven to be an efficient ferroptosis/apoptosis hybrid anti-cancer agent. This strategy may provide new method for improving the clinical outcomes of traditional cancer therapies.

## Materials and methods

### Synthesis of BSO@MOF

1 mL FeCl_3_⋅6H_2_O in ethanol (27 mg mL^−1^) and 200 μL pure water were rapidly added to 2-amino-terephthalic acid ethanol solution under 1200 rpm magnetic stirring. After magnetic stirring for 10 min, the mixture was incubated at 50 ℃ thermostatic water baths for 1 h followed by centrifuging at 12000 rpm for 15 min to obtain MOF. Then, added proper concentration of BSO aqueous solution dropwise into the 4 mL of MOF ethanol solution slowly under the ultrasound. Subsequently, the mixture was shocked overnight, and then centrifuged and dissolved in 1 mL ethanol for later use.

### Lipid bilayer modification of BSO@MOF

The BSO@MOF was encapsulated with lipid bilayer using a two-step approach. First, 4 mL chloroform, 75 μL DOPC (25 mg mL^−1^) and 1 mL BSO@MOF (1 mg mL^−1^) in ethanol were added to a pear-shaped flask and sonicated for 15 min. After drying, 4 mL chloroform was added again and sonicated for 30 min followed by drying. Second, 2 mL chloroform, 50 μL DOPC (25 mg mL^−1^), 25 μL cholesterol, and 100 uL PEG-2000 PE (25 mg mL^−1^) were added and sonicated for 15 min. After the spin dry, the mixture dried overnight in a vacuum drying oven. Lastly, added 4 mL purified water and sonicated for 30 min, then centrifuged and dissolved in 1 mL deionized water to obtain BSO@MOF-L aqueous solution.

### Lipid bilayer and RGD dual modification of BSO@MOF

For in vivo experiments, lipid bilayer and RGD double modification of BSO@MOF is required. Briefly, sulfo-SMCC and 1,2-dioleoyl-sn-glycero-3-phosphoethanolamine (DOPE) was dissolved in DMSO respectively, then were mixed and incubated at room temperature for 30 min to obtain DOPE-SMCC. Subsequently, c (RGDfC) polypeptide was dissolved in DMSO and then added to DOPE-SMCC. After stirring at 4 ℃ for 2 h, excess SMCC was removed by a desalting column and freeze-dried, then dissolved in chloroform to obtain DOPE-RGD.

Afterward, BSO@MOF-L in ethanol was mixed with DOPC in chloroform under sonication for 15 min. Subsequently, the mixture was dried and washed with chloroform to remove uncapped free DOPC and then redispersed in chloroform solution that containing DOPC, cholesterol, PEG-2000 PE, and DOPE-RGD under sonicated for 15 min. After the spin dry, the mixture dried overnight in a vacuum drying oven. Lastly, added 4 mL purified water and sonicated for 30 min, then centrifuged and dissolved in 1 mL deionized water to obtain BSO@MOF-LR aqueous solution for later use.

### Cell culture

4T1 murine breast cancer cells were cultured in RMPI 1640 medium containing 10% FBS and 1% antibiotics (penicillin/streptomycin, 10 000 U mL^−1^) at 37 °C under 5% CO_2_. HEK-293 cells were incubated in DMEM medium supplemented with 10% FBS and 1% antibiotics at 37 °C under 5% CO_2_.

### In vitro cytotoxicity evaluation

The 4T1 cells were seeded in 96-well plates with the density of 7 × 10^3^ cells per well and incubated for 12 h. Then BSO, MOF-L, or BSO@MOF-L with different concentrations (25, 50, and 100 μg mL^−1^) were added and co-cultured with cells for 24 h. To evaluate cell viability, 20 μL of 3-(4,5-dimethylthiazol-2-yl)-2,5-diphenyltetrazolium bromide (MTT) (5 mg mL^−1^) solution was added into each well and incubated at 37 °C for 4 h. Finally, 150 μL of DMSO was added to each well and the formazan absorbance was detected at 492 nm by using a multiskan. Data were presented as average ± SD (n = 4).

### Cellular GSH assay

4T1 cells were seeded in 6-well plates at a density of 2 × 10^5^ per well. After 12 h, cells were treated with PBS, BSO, MOF-L, or BSO@MOF-L for 12 h. Then, the cells were harvested to measure GSH content using a total glutathione assay kit (Beyotime, S0052) according to the manufacturer’s instructions. The percentage content of GSH was acquired based on the comparison to the GSH content of untreated cells.

### GPX4 activity and expression analysis

In brief, 4T1 cells were seeded in 6-well plates and cultured for 12 h before use. After various treatments for 12 h, the activity of GPX4 in 4T1 cells was determined using the cellular glutathione peroxidase assay kit (Beyotime, S0056) with reference to the product protocol. Similarly, the expression of GPX4 in 4T1 cells upon different treatments was also analyzed by western blotting. The cell lysates containing identical protein (20 µg) were separated by SDS-PAGE and transferred to membrane, followed by antibody incubation. The dilution ratio for the first antibody was 1:2000 (anti-GPX4) and 1:20000 (anti-β-actin), the dilution ratio was 1:1000 for the secondary antibody.

### Cellular DCFH-DA assay

The generated ROS was measured by microscopy using an ROS-sensitive probe DCFH-DA (Beyotime). 4T1 cells were seeded in 24-well plates or 12-well plates and cultured at 5% CO_2_, 37 °C overnight. Drugs were added at concentration of 100 μg mL^−1^ and cultured for 12 h. Then the cells were stained by DCFH-DA (10 μM) and incubated for 30 min. The microscopy (FV 1200, Olympus, Tokyo, Japan) was used to obtain the fluorescence images of cells and flow cytometry was used to quantitative analysis.

### Intracellular LPO generation

BODIPY581/591-C11 (Thermo Fisher), a fluorescent probe for LPO detection that can insert into lipid membranes and been oxidized by intracellular LPO, was used to assess the intracellular LPO level. In brief, 4T1 cells were seeded in 24-well plates or 12-well plates and incubated for 12 h. Then the cells were added with drugs and incubated for another 12 h. After that, the cells were incubated in the serum-free RMPI 1640 medium containing BODIPY581/591-C11 (10 μM) for 30 min. After being washed with PBS, the cells were subjected to microscopy observation or flow cytometry analysis.

### In vitro cellular uptake assay

The 4T1 cells were seeded in 24-well plates or 12-well plates and incubated for 12 h. Then MOF-L or MOF-LR were added into each well, and the cells were incubated for another 6 h. After that, the cells were washed by PBS before the observation by microscopy and quantitative analysis by flow cytometry.

### Tumor model construction

BALB/c female mice (4–5 weeks old) were supplied by the Animal Center of the Fourth Military Medical University (FMMU). All procedures were approved by the Institutional Animal Care and Use Committee of the Fourth Military Medical University (Xi’an, China). The tumor model was established by subcutaneous injection of 1 × 10^7^ 4T1 cells in 200 μL PBS per mouse. The tumor volume was allowed to grow to 100 mm^3^ for further use.

### In vivo tumor inhibition

The 4T1 tumor-bearing BALB/c mice were divided randomly into seven groups and intravenously injected with PBS, MOF-LR, free OXA, OXA@MOF-LR, BSO@MOF-LR, and BSO&OXA@MOF-LR every two days to a total of six times. In the ferroptosis inhibition group BSO&OXA@MOF-LR + DFO, ferroptosis inhibitor DFO was administrated via intraperitoneal injection. Body weights and tumor volumes were measured every 2 days during the treatment. After 12 days, the mice were sacrificed, and the tumor were harvested for H&E staining.

### Supplementary Information

**Additional file 1: Fig. S1** The cell viability of 4T1 cells after treated with different concentrations of BSO@MOF-L at different time points. **Fig. S2** Cell viability of 4T1 cells after treated with BSO@MOF-L and different concentrations of apoptosis inhibitor Z-VAD-FMK. **Fig.S3 **GPX4 activity of 4T1 cells after treatment with PBS, BSO, MOF-L, or BSO@MOF-L. **Fig. S4 **Characterization of the BSO&OXA@MOF-LR nanoparticles. Hydrodynamic size (a) and zeta potential (b) of MOF, BSO&OXA@MOF, and BSO&OXA@MOF-LR (n=3). **Fig. S5** The cell viability of 4T1 cells after different treatments with various concentrations (n=5). **Fig. S6** Individual tumor growth kinetics in mice with different treatments. **Fig. S7** Body weight of 4T1-tumor-bearing mice with different treatments. **Fig. S8 **H&E staining indicated minimal lesions to heart, liver, spleen, lung and kidney both in Ctrl (G1) and BSO&OXA@MOF-LR (G7) groups. Scale bar: 500 μm.

## Data Availability

All data generated or analyzed during this study are included in this published article.
