# Supplementary material for: Iron-based metal-organic framework co-loaded with buthionine sulfoximine and oxaliplatin for enhanced cancer chemo-ferrotherapy via sustainable glutathione elimination
Source: J Nanobiotechnology. 2023 Aug 10;21:265. doi: 10.1186/s12951-023-01998-w (PMC10416514; doi:10.1186/s12951-023-01998-w)
Supplement: Supplementary file 1 — Additional file 1: Fig. S1 The cell viability of 4T1 cells after treated with different concentrations of BSO@MOF-L at different time points. Fig. S2 Cell viability of 4T1 cells after treated with BSO@MOF-L and different concentrations of apoptosis inhibitor Z-VAD-FMK. Fig.S3 GPX4 activity of 4T1 cells after treatment with PBS, BSO, MOF-L, or BSO@MOF-L. Fig. S4 Characterization of the BSO&OXA@MOF-LR nanoparticles. Hydrodynamic size (a) and zeta potential (b) of MOF, BSO&OXA@MOF, and BSO&OXA@MOF-LR (n=3). Fig. S5 The cell viability of 4T1 cells after different treatments with various concentrations (n=5). Fig. S6 Individual tumor growth kinetics in mice with different treatments. Fig. S7 Body weight of 4T1-tumor-bearing mice with different treatments. Fig. S8 H&E staining indicated minimal lesions to heart, liver, spleen, lung and kidney both in Ctrl (G1) and BSO&OXA@MOF-LR (G7) groups. Scale bar: 500 μm. [file 12951_2023_1998_MOESM1_ESM.docx]

# Additional file

**Iron-Based Metal-Organic Framework Co-loaded with Buthionine Sulfoximine and Oxaliplatin for Enhanced Cancer Chemo-ferrotherapy via Sustainable Glutathione Elimination**

*Zhiping Rao, Yutian Xia, Qian Jia, Yutong Zhu,* *Lexuan Wang, Guohuan Liu, Xuelan Liu, Peng Yang, Pengbo Ning, Ruili Zhang, Xianghan Zhang, Chaoqiang Qiao^*^, Zhongliang Wang^*^*

This additional file only amended the title.

**Fig. S1** The cell viability of 4T1 cells after treated with different concentrations of BSO@MOF-L at different time points.

**Fig. S2** Cell viability of 4T1 cells after treated with BSO@MOF-L and different concentrations of apoptosis inhibitor Z-VAD-FMK.


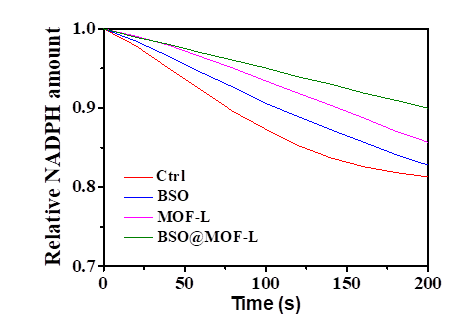


**Fig.S3** GPX4 activity of 4T1 cells after treatment with PBS, BSO, MOF-L, or BSO@MOF-L.


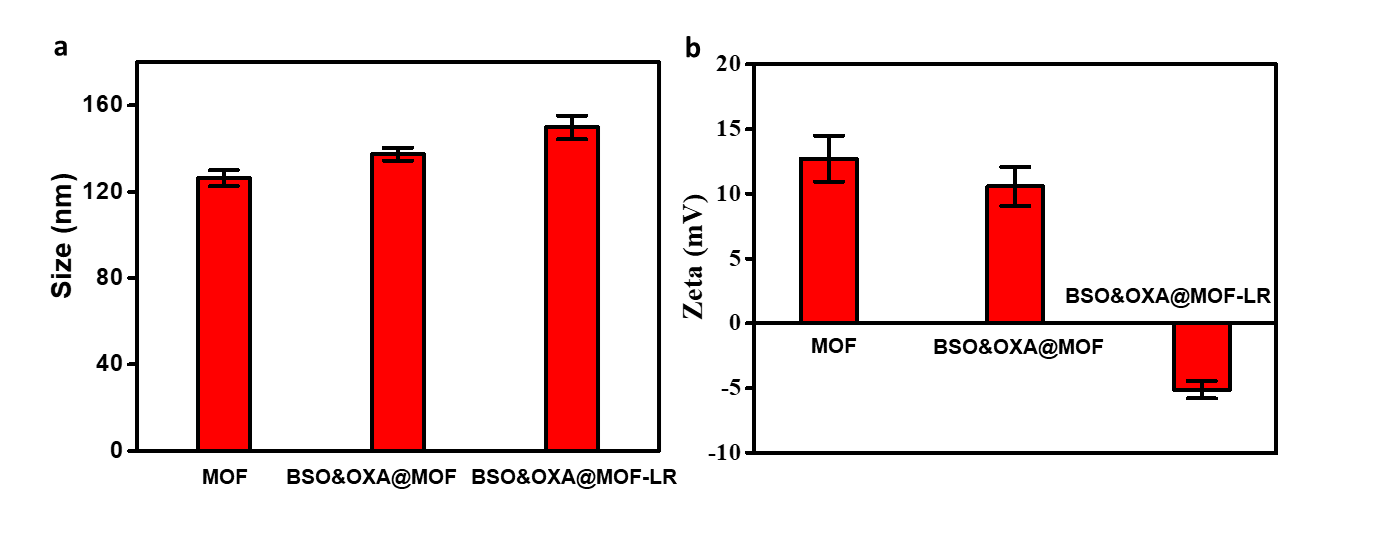


**Fig. S4** Characterization of the BSO&OXA@MOF-LR nanoparticles. Hydrodynamic size (a) and zeta potential (b) of MOF, BSO&OXA@MOF, and BSO&OXA@MOF-LR (n=3).

**Fig. S5** The cell viability of 4T1 cells after different treatments with various concentrations (n=5).


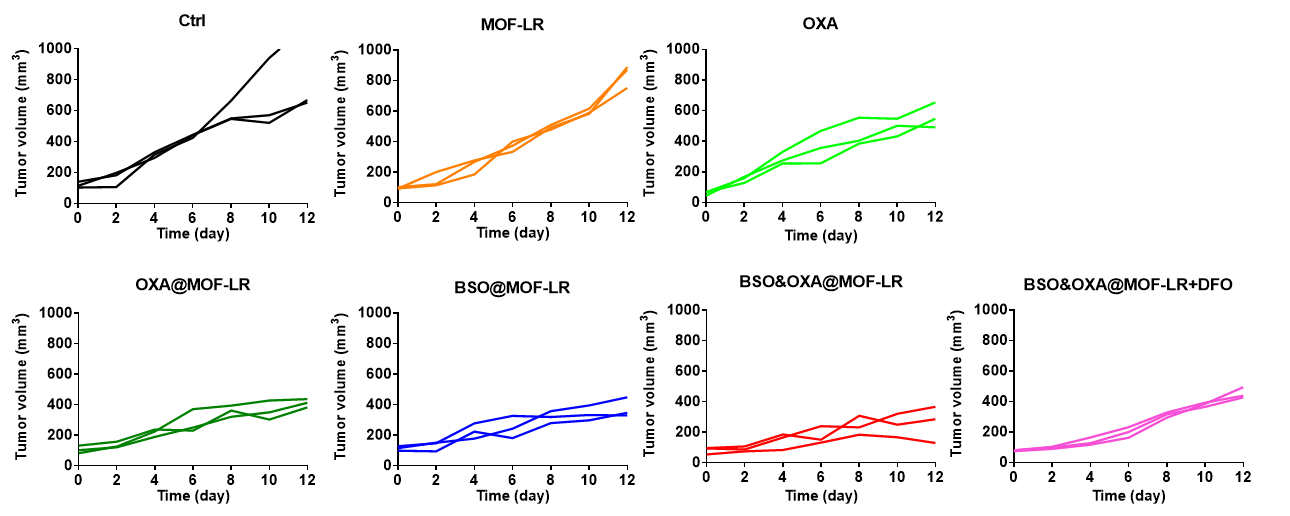


**Fig. S6** Individual tumor growth kinetics in mice with diﬀerent treatments.

**Fig. S7** Body weight of 4T1-tumor-bearing mice with different treatments.


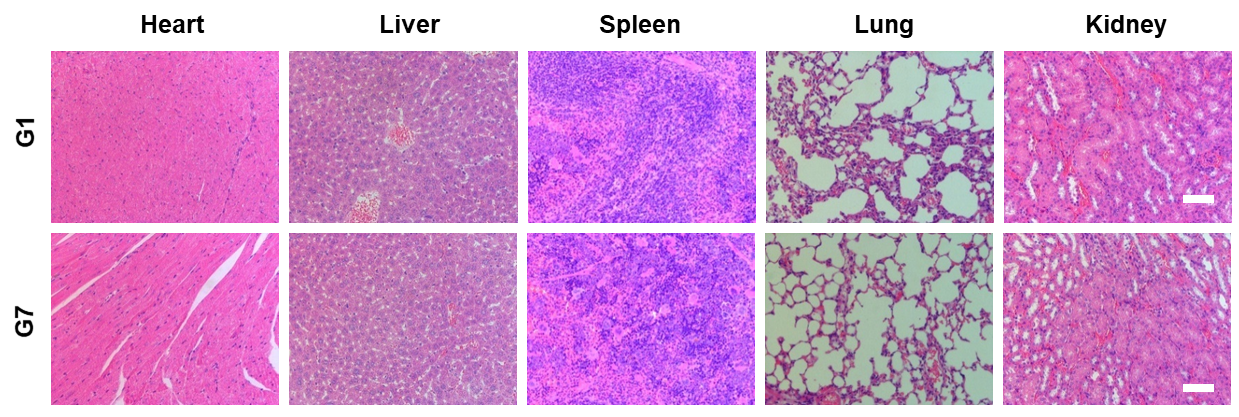


**Fig. S8** H&E staining indicated minimal lesions to heart, liver, spleen, lung and kidney both in Ctrl (G1) and BSO&OXA@MOF-LR (G7) groups. Scale bar: 500 μm.
